# Supplementary material for: Examining the Role of Interpersonal Violence in Racial Disparities in Breastfeeding in North Dakota (ND PRAMS 2017–2019)
Source: Int J Environ Res Public Health. 2023 Apr 9;20(8):5445. doi: 10.3390/ijerph20085445 (PMC10138366; doi:10.3390/ijerph20085445)
Supplement: Supplementary file 1 [file ijerph-20-05445-s001.zip › ijerph-2284838-supplementary.pdf]

International Journal of
Environmental Research and Public Health

Table S1. Education variable removed from main breastfeeding initiation analyses.

		Breastfeeding Initiation OR (95% CI)			
		American Indian	Other Racial Identities	White	Violence Variable Estimate
**Crude Model**		0.24 (0.18, 0.31)	0.87 (0.49, 1.52)	Ref.	-
**Sociodemographic Model**		0.55 (0.37, 82)	1.09 (0.58, 2.06)	Ref.	-
**Violence Before Pregnancy**	**Any Interpersonal Violence**	0.54 (0.37, 0.81)	1.11 (0.59, 2.08)	Ref.	0.36 (0.10, 1.28)
	**Husband/Partner**	0.55 (0.37, 0.82)	1.09 (0.58, 2.06)	Ref.	0.95 (0.37, 2.42)
	**Ex- Husband/Partner**	0.55 (0.37, 0.82)	1.09 ((0.58, 2.06)	Ref.	0.97 (0.35, 2.66)
	**Other Family**	0.56 (0.37, 0.83)	1.10 (0.58, 2.06)	Ref.	0.65 (0.22, 1.89)
	**Other**	0.55 (0.37, 0.81)	1.09 (0.58, 2.06)	Ref.	0.46 (0.18, 1.20)
**Violence During Pregnancy**	**Any Interpersonal Violence**	0.55 (0.37, 0.82)	1.09 (0.58, 2.07)	Ref.	0.66 (0.18, 2.34)
	**Husband/Partner**	0.54 (0.36, 0.81)	1.08 (0.57, 2.05)	Ref.	1.34 (0.52, 3.47)
	**Ex- Husband/Partner**	0.55 (0.37, 0.81)	1.09 (0.58, 2.06)	Ref.	1.41 (0.46, 4.37)
	**Other Family**	0.55 (0.37, 0.82)	1.09 (0.58, 2.06)	Ref.	0.85 (0.33, 2.19)
	**Other**	0.55 (0.37, 0.81)	1.09 (0.58, 2.06)	Ref.	1.23 (0.42, 3.59)

Adjusted for race, income, insurance before pregnancy, maternal age, substance use, marital status, pregnancy intention, chronic disease, overweight/obese, WIC, Kotelchuck, and ACEs.

Table S2. Income variable removed from main breastfeeding initiation analyses.

		Breastfeeding Initiation OR (95% CI)			
		**American Indian**	**Other Racial Identities**	**White**	**Violence Variable Estimate**
**Crude Model**		0.24 (0.18, 0.31)	0.87 (0.49, 1.52)	Ref.	-
**Sociodemographic Model**		0.52 (0.35, 0.78)	1.02 (0.54, 1.93)	Ref.	-
**Violence Before Pregnancy**	**Any Interpersonal Violence**	0.52 (0.35, 0.77)	1.04 (0.55, 1.97)	Ref.	0.38 (0.10, 1.36)
	**Husband/Partner**	0.52 (0.35, 0.78)	1.02 (0.54, 1.93)	Ref.	0.94 (0.37, 2.36)
	**Ex- Husband/Partner**	0.52 (0.35, 0.78)	1.02 (0.54, 1.93)	Ref.	0.94 (0.35, 2.47)
	**Other Family**	0.53 (0.35, 0.79)	1.03 (0.54, 1.94)	Ref.	0.67 (0.23, 1.92)
	**Other**	0.52 (0.35, 0.78)	1.03 (0.54, 1.94)	Ref.	0.48 (0.8, 1.31)
**Violence During Pregnancy**	**Any Interpersonal Violence**	0.52 (0.35, 0.78)	1.02 (0.54, 1.94)	Ref.	0.77 (0.20, 2.85)
	**Husband/Partner**	0.52 (0.34, 0.77)	1.02 (0.54, 1.92)	Ref.	1.37 (0.53, 3.50)
	**Ex- Husband/Partner**	0.52 (0.35, 0.78)	1.02 (0.54, 1.93)	Ref.	1.30 (0.45, 3.69)
	**Other Family**	0.52 (0.35, 0.78)	1.02 (0.54, 1.93)	Ref.	0.86 (0.34, 2.21)
	**Other**	0.52 (0.35, 0.78)	1.02 (0.54, 1.93)	Ref.	1.26 (0.43, 3.71)

Adjusted for race, insurance before pregnancy, maternal age, education, substance use, marital status, pregnancy intention, chronic disease, overweight/obese, WIC, Kotelchuck, and ACEs.

Table S3. Education variable removed from main 2 month breastfeeding duration analyses.

		Breastfeeding Duration – 2 Months OR (95% CI)			
		**American Indian**	**Other Racial Identities**	**White**	**Violence Variable Estimate**
**Crude Model**		0.30 (0.25, 0.36)	1.08 (0.75, 1.57)	Ref.	-
**Sociodemographic Model**		0.70 (0.53, 0.93)	1.23 (0.76, 1.99)	Ref.	-
**Violence Before Pregnancy**	**Any Interpersonal Violence**	0.70 (0.52, 0.93)	1.25 (0.77, 2.02)	Ref.	0.35 (0.07, 1.56)
	**Husband/Partner**	0.72 (0.54, 0.96)	1.24 (0.77, 2.01)	Ref.	0.60 (0.23, 1.53)
	**Ex- Husband/Partner**	0.70 (0.53, 0.94)	1.24 (0.77, 2.00)	Ref.	0.69 (0.30, 1.60)
	**Other Family**	0.72 (0.54, 0.96)	1.24 (0.77, 2.01)	Ref.	0.44 (0.13, 1.41)
	**Other**	0.70 (0.53, 0.93)	1.24 (0.77, 1.99)	Ref.	0.47 (0.18, 1.18)
**Violence During Pregnancy**	**Any Interpersonal Violence**	0.71 (0.53, 0.94)	1.25 (0.77, 2.03)	Ref.	0.22 (0.05, 1.02)
	**Husband/Partner**	0.72 (0.54, 0.96)	1.25 (0.78, 2.03)	Ref.	0.50 (0.20, 1.24)
	**Ex- Husband/Partner**	0.71 (0.53, 0.94)	1.24 (0.77, 2.00)	Ref.	0.47 (0.18, 1.22)
	**Other Family**	0.72 (0.54, 0.96)	1.24 (0.77, 2.00)	Ref.	0.50 (0.16, 1.53)
	**Other**	0.70 (0.53, 0.94)	1.23 (0.76, 1.99)	Ref.	0.60 (0.22, 1.58)

Adjusted for race, income, insurance before pregnancy, maternal age, substance use, marital status, pregnancy intention, chronic disease, overweight/obese, WIC, Kotelchuck, and ACEs.

Table S4. Income variable removed from main 2 month breastfeeding duration analyses.

		Breastfeeding Duration – 2 Months OR (95% CI)			
		**American Indian**	**Other Racial Identities**	**White**	**Violence Variable Estimate**
**Crude Model**		0.30 (0.25, 0.36)	1.08 (0.75, 1.57)	Ref.	-
**Sociodemographic Model**		0.68 (0.51, 0.90)	1.18 (0.73, 1.89)	Ref.	-
**Violence Before Pregnancy**	**Any Interpersonal Violence**	0.68 (0.51, 0.90)	1.19 (0.74, 1.92)	Ref.	0.35 (0.08, 1.52)
	**Husband/Partner**	0.70 (0.52, 0.93)	1.19 (0.74, 1.91)	Ref.	0.59 (0.23, 1.48)
	**Ex- Husband/Partner**	0.68 (0.51, 0.90)	1.18 (0.74, 1.91)	Ref.	0.68 0.28, 1.59)
	**Other Family**	0.69 (0.52, 0.92)	1.19 0.74, 1.91)	Ref.	0.44 (0.14, 1.39)
	**Other**	0.68 (0.51, 0.90)	1.18 (0.73, 1.90)	Ref.	0.47 (0.18, 1.20)
**Violence During Pregnancy**	**Any Interpersonal Violence**	0.68 (0.51, 0.91)	1.19 (0.74, 1.92)	Ref.	0.23 (0.05, 1.09)
	**Husband/Partner**	0.69 (0.52, 0.92)	1.19 (0.74, 1.92)	Ref.	0.50 (0.20, 1.25)
	**Ex- Husband/Partner**	0.69 (0.52, 0.91)	1.18 (0.74, 1.90)	Ref.	0.44 (0.17, 1.14)
	**Other Family**	0.69 (0.52, 0.92)	1.18 (0.74, 1.90)	Ref.	0.49 (0.16, 1.49)
	**Other**	0.68 (0.51, 0.90)	1.18 (0.73, 1.89)	Ref.	0.59 (0.22, 1.55)

Adjusted for race, insurance before pregnancy, maternal age, education, substance use, marital status, pregnancy intention, chronic disease, overweight/obese, WIC, Kotelchuck, and ACEs.

Table S5. Education variable removed from main 6 month breastfeeding duration analyses.

		Breastfeeding Duration – 6 Months OR (95% CI)			
		**American Indian**	**Other Racial Identities**	**White**	**Violence Variable Estimate**
**Crude Model**		0.24 (0.20, 0.29)	0.87 (0.62, 1.23)	Ref.	-
**Sociodemographic Model**		0.62 (0.46, 0.83)	0.88 (0.56, 1.36)	Ref.	-
**Violence Before Pregnancy**	**Any Interpersonal Violence**	0.62 (0.46, 0.83)	0.88 (0.57, 1.37)	Ref.	0.58 (0.11, 3.00)
	**Husband/Partner**	0.63 (0.47, 0.84)	0.88 (0.56, 1.37)	Ref.	0.69 (0.24, 1.98)
	**Ex- Husband/Partner**	0.62 (0.46, 0.83)	0.88 (0.56, 1.37)	Ref.	0.47 (0.18, 1.20)
	**Other Family**	0.63 (0.47, 1.37)	0.88 (0.57, 1.37)	Ref.	0.47 (0.12, 1.76)
	**Other**	0.62 (0.46, 0.83)	0.88 (0.56, 1.36)	Ref.	0.69 (0.25, 1.93)
**Violence During Pregnancy**	**Any Interpersonal Violence**	0.62 (0.47, 0.83)	0.89 (0.57, 1.38)	Ref.	0.33 (0.06, 1.72)
	**Husband/Partner**	0.63 (0.47, 0.84)	0.89 (0.57, 1.38)	Ref.	0.54 (0.20, 1.43)
	**Ex- Husband/Partner**	0.62 (0.47, 0.83)	0.88 (0.56, 1.36)	Ref.	0.74 (0.26, 2.11)
	**Other Family**	0.63 (0.47, 0.84)	0.88 (0.56, 1.37)	Ref.	0.70 (0.20, 2.45)
	**Other**	0.62 (0.47, 0.83)	0.88 (0.56, 1.36)	Ref.	0.65 (0.23, 1.81)

Adjusted for race, income, insurance before pregnancy, maternal age, substance use, marital status, pregnancy intention, chronic disease, overweight/obese, WIC, Kotelchuck, and ACEs.

Table S6. Income variable removed from main 6 month breastfeeding duration analyses.

		Breastfeeding Duration – 6 Months OR (95% CI)			
		**American Indian**	**Other Racial Identities**	**White**	**Violence Variable Estimate**
**Crude Model**		0.24 (0.20, 0.29)	0.87 (0.62, 1.23)	Ref.	-
**Sociodemographic Model**		0.58 (0.44, 0.77)	0.79 (0.51, 1.22)	Ref.	-
**Violence Before Pregnancy**	**Any Interpersonal Violence**	0.58 (0.43, 0.77)	0.79 (0.51, 1.23)	Ref.	0.58 (0.12, 2.80)
	**Husband/Partner**	0.59 (0.44, 0.78)	0.79 (0.51, 1.23)	Ref.	0.67 (0.24, 1.85)
	**Ex- Husband/Partner**	0.58 (0.43, 0.77)	0.79 (0.51, 1.23)	Ref.	0.45 (0.18, 1.13)
	**Other Family**	0.59 (0.44, 0.79)	0.79 (0.51, 1.23)	Ref.	0.46 (0.13, 1.65)
	**Other**	0.58 (0.43, 0.77)	0.79 (0.51, 1.22)	Ref.	0.69 (0.24, 1.92)
**Violence During Pregnancy**	**Any Interpersonal Violence**	0.58 (0.44, 0.77)	0.80 (0.51, 1.23)	Ref.	0.36 (0.06, 1.90)
	**Husband/Partner**	0.59 (0.44, 0.78)	0.80 (0.52, 1.24)	Ref.	0.54 (0.20, 1.45)
	**Ex- Husband/Partner**	0.58 (0.44, 0.77)	0.79 (0.51, 1.22)	Ref.	0.67 (0.24, 1.87)
	**Other Family**	0.59 (0.44, 0.78)	0.79 (0.51, 1.22)	Ref.	0.69 (0.21, 2.32)

Adjusted for race, insurance before pregnancy, maternal age, education, substance use, marital status, pregnancy intention, chronic disease, overweight/obese, WIC, Kotelchuck, and ACEs.
